# Farmers’ indigenous knowledge on local herbaceous forages in the Northeastern highlands of Ethiopia

**DOI:** 10.1371/journal.pone.0352188

**Published:** 2026-06-26

**Authors:** Hussen Ebrahim, Yeshambel Mekuriaw, Bimrew Asmare, Netsanet Beyero, Fentahun Meheret Zeleke, Shewaye Hailecherkos, Endalew Mekonen, Zerihun Nigussie, Wubetie Adnew

**Affiliations:** 1 Department of Animal Science, College of Agriculture, Woldia University, Woldia, Amhara Regional State, Ethiopia; 2 Department of Animal Science, College of Agriculture and Environmental Science, Bahir Dar University, Bahir Dar, Amhara Regional State, Ethiopia; 3 Department of Agricultural Economics, College of Agriculture and Environmental Science, Bahir Dar University, Bahir Dar, Amhara Regional State, Ethiopia; 4 Department of Boilogy, College of Science, Bahir Dar University, Bahir Dar, Amhara Regional State, Ethiopia; PLOS: Public Library of Science, UNITED STATES OF AMERICA

## Abstract

Natural pasture (NP) occupies the top place in livestock feed, particularly in frost-affected highland areas of Ethiopia. Despite numerous studies reporting the huge contribution of local herbaceous forages (LHF) in livestock production, the implementation of those results was not fruitful, attributable to overlooking farmers’ knowledge and experience. Therefore, the study was carried out to scrutinize the indigenous knowledge on LHF in the NP of the northeastern Highlands of Ethiopia. In the present study, we use 323 smallholder farmers (SHF) selected using systematic random sampling from two purposively selected Districts (Mekidela and Tenta) and eight Kebeles employing a multistage sampling procedure. The study analyzed the primary data and highlighted the relevance of SHF’s deep-rooted indigenous knowledge to improving NP and LHF. The findings confirmed that livestock production is mainly dependent on NP in the study area. However, farmers perceived that the NP has been declining with time at an alarming rate, mainly due to forestland (index (*I)* = 0.456), cropland (*I* = 0.338), and resettlement (*I* = 0.139) expansion. The study elucidated that land shortage (*I* = 0.172), lack of awareness (*I* = 0.17), and eucalyptus expansion (*I* = 0.151) were the first, second, and third ranked challenges of utilizing LHF. Farmers reduced animal numbers (*I* = 0.234) and practiced zero grazing (*I* = 0.34) to improve the LHF in the NP during the dry and wet seasons, respectively. Moreover, further investigations are required to elucidate more merits of LHF species, determine their species diversity, and evaluate their morphological characters.

## Introduction

Ethiopia is largely dependent on natural resources for subsistence [1] and faces devastating phenomena such as flooding, drought, conflict, and food insecurity [2,3]. Accordingly, the livestock sector has a multifunctional advantage under severe conditions. In Ethiopia, the livestock population is increasing at an alarming rate, but in the meantime, the pastureland is shrinking. FAO [4] reported that the country’s livestock feed demand reached approximately 130 million tons of dry matter, which was far beyond the annual feed supply [5,4,6]. As a result, livestock owners experience feed shortages during the wet (50%) and dry (84%) seasons of the year [7]. The feed shortage is because of not only to low production but also to the utilization of feed resources for other purposes, such as housing [8]. However, the availability of nutritionally poor feed is the first and foremost impediment in livestock production, and the problem is getting worse with time [5,9,10].

Natural pasture constitutes the largest portion of livestock feed in Ethiopia [11], sharing a huge contribution in the highland parts of the country, particularly in frost-affected areas [12]. Ethiopia is not utilizing the natural pasture up to its maximum potential to produce the required feed (both in quantity and quality) pertaining to different factors. Climate change [13,14] and overgrazing [15–17] have an enormous impact on the country’s natural pastures following high population pressure [18]. Low and unreliable rainfall [19–21] hinders the production potential of natural pasture. In addition, conversion into cropland and bush-land [22], soil erosion [23], and weeds [8] have shrunk natural pasture. The worst news is that degraded pastureland requires a longer time to recover [24]. It is apparent that the expansion of cropland has resulted in a devastating contraction of natural pasture by 18.1% [22,25] to more than 50% [26]. In addition, eucalyptus tree plantations reduced both the size and productivity of natural pasture, particularly in the highland parts of the country [27].

Natural pasture sustains a higher number of livestock populations as feed and/or nutrient source in the highlands of Ethiopia, which caused land degradation [17] and biodiversity loss [28,29]. Accordingly, appropriate pastureland management and utilization enable the adjustment of grazing intensity to increase productivity and modify species composition [30]. This can be achieved by stopping free grazing [31–33], adjusting defoliation intensity [34–37], applying farmyard manure, and planting legumes [38].

Despite a number of research outputs reporting on the contribution, species composition, biomass, and nutritional content of forage species in different parts of Ethiopia, the implementation of those results was not fruitful, as the result of overlooking farmers’ knowledge and experience [39]. Moreover, there are limited studies on exploiting the indigenous knowledge at the smallholder level under the dynamic change of natural pasture. The indigenous knowledge of farmers is foundational to setting selection criteria and identifying potential feed resources [40], which then ease the implementation of successful attempts [41]. In this regard, previous works reported promising results by actively participating farmers in their study process [42,43], though their work is limited only to certain localities. In addition, Atsbha et al. [44] recommended the integral role of local people, researchers, and government bodies to ensure sustainable effort in improving the species composition and productivity of natural pasture, particularly communal pasturelands. Even though some attempts were made to evaluate the indigenous knowledge related to natural pasture in some parts of the country [45,46], there was no information in the Northeastern highlands of Ethiopia despite this area occupies a large land mass that maintains huge livestock population in the nation. Therefore, the study was carried out to scrutinize the indigenous knowledge on local herbaceous forages in the natural pastureland of the Northeastern highlands of Ethiopia, particularly Tenta and Mekidela Districts.

## Materials and methods

### Description of study areas

The study included two purposively selected Districts of the Northeastern highlands of Ethiopia. The Districts’ elevation ranges between 700 and 4100 m above sea level. Mekdela and Tenta Districts are situated about 537 and 520 km north of the capital city, Addis Ababa, and 137 and 120 km west of Dessie town, respectively. Tenta District receives an annual rainfall range between 592 mm and 1216 mm, of which 75% of the rain occurs in the wet season [47]. Mekdela District gets annual rainfall range between 495 mm and 1168 mm [48]. Overall, the highlands of northeastern Ethiopia predominantly have an annual temperature and rainfall ranging from 12°C to 28°C and 800 mm to 1200 mm, respectively, with an increase in annual temperature of 0.07°C [49–51]. The mean monthly rainfall pattern shows that the Northeastern highlands of Ethiopia are in their wet season from June to September [52]. The Districts have temperature ranges between 6.4 and 22°C with a mean value of 13.6°C [47,48].

### Sampling and sample size determination

The study had an initial rapid survey to get baseline information on the knowledge of experts and smallholder farmers and to establish a sampling framework from which samples were taken. That rapid field survey was undertook to understand the socio-economic attributes of farmers, indigenous knowledge on feed resource availability, and natural pasture status, utilization practices, and management. Then, the study employed a multistage sampling procedure: two Districts (Tenta and Mekdela) from the Northeastern highlands of Ethiopia were purposively selected based on their security status, livestock population, natural pasture, and access to sampling.

Discussion with each District livestock and fishery office expert resulted in the purposive selection of eight Kebeles: Deferge, Kibtiya, Genatit, and Dedere from Mekidela District and Fitto, Kurkur, Yamed, and Cheleme from Tenta out of 62 rural kebeles, considering the aforesaid criteria. Then, the study employed systematic random sampling to select SHF every 8^th^ interval of their list in each Kebele. The selected SHF had livestock and had extensive experience in natural pasture and indigenous herbaceous forage (IHF) utilization and management, and administered a questionnaire interview. In addition, focus group discussions (FGDs) were held with experts at each District and experienced farmers and development agents at each Kebele to clarify issues that had not been well collected in the questionnaire survey. Overall, the present study addressed 323 smallholder farmers selected based on the Cochran formula [53] with a 95% confidence level.


$$\begin{array}{c} no=\frac{Z^{\text{2}}}{e^{\text{2}}}pq \end{array}$$ (1)

Where, no = desired sample size according to Cochran when population is greater than 10,000; p = the estimated proportion of the population who has grazing land or pasture land (0.30), q = 1 – p = 0.70, e = the desired level of precision or the margin of error (0.05)


$$\begin{array}{c} no=\frac{{\text{1}.\text{9}\text{6}}^{\text{2}}}{{\text{0}.\text{0}\text{5}}^{\text{2}}}*\text{0}.\text{3}*\text{0}.\text{7}=\text{ 322}.\text{69 }\approx \text{ 323} \end{array}$$ (2)

**Fig 1:**
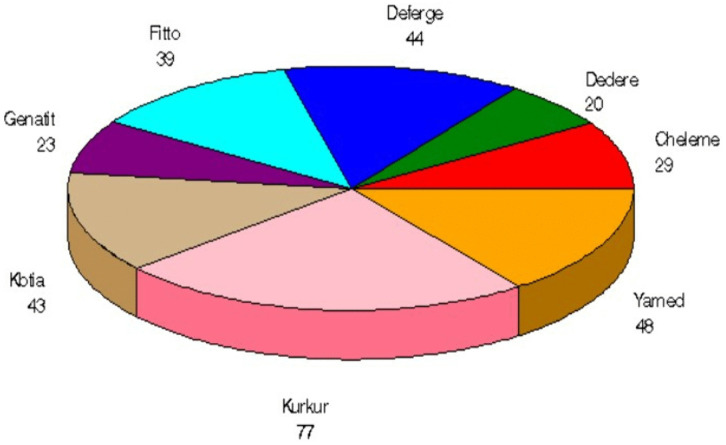
Distribution of sample population to each Kebele.


Then, the total sample size was distributed to each Kebele using the proportional sampling method with the following formula, as illustrated in Fig 1.

$$\begin{array}{c} n\text{1}=\frac{nN\text{1}}{N} \end{array}$$ (3)

where n is the total number of samples, N is the total number of household heads that had livestock, N1 is the number of household heads that had livestock in each Kebele, and n1 is the number of samples from each Kebele.

The questionnaire was structured and validated. It covers socio-economic attributes, trends in livestock population and reasons, the predominant land use types, and the status, utilization practices, management, challenges, and mitigation options of natural pasture and IHF species. The questionnaire was translated from English into Amharic, and the structured and semi-structured questions were pretested by 20 SHF who were not included in the actual study. Then, some improvements were made. Eight enumerators were oriented and trained on questionnaire administration and response registration. The questionnaires were coded from 1 to 323 before the actual investigation and distributed in sequence to enumerators to easily recognize who managed the questionnaire. Each interview was conducted in different settings, such as at natural pastures where smallholder farmers herd animals, homes, soil and water conservation sites, and croplands. The responses were translated from Amharic into English immediately after the completion of response data collection.

### Ethics statement

The respondents included in this study provided verbal consent to the researchers, which was documented through audio recordings and cross-verified using written confirmation in the interview questionnaire. This served as both proof of agreement and a record of the consent process for using the collected data for scientific purposes and anonymous publication. A designated witness was present during each recording to confirm that consent was given voluntarily and with full understanding of the research’s purpose and methodology. We used the verbal agreement because many of our participants were illiterate and could not read or understand a written consent form. The first author documented participants’ consent in a secure study log immediately after each interview. A neutral third-party witness was present during the consent process to confirm that participants were fully informed and voluntarily agreed to participate. This procedure was approved by the Research Ethics Review Committee (RERC) of the College of Agriculture and Environmental Sciences at Bahir Dar University, which granted a waiver of written consent (Approval Reference No. 7/002/BDU.1500156).

### Data collection

Data on household characteristics, livestock holdings, herd structure, major feed resources, and the status, utilization practices, challenges, and mitigation options of natural pasture, particularly indigenous herbaceous grass and legume species (IHGLS) were collected from farmers through eighteen structured and semi-structured questions. The investigators coded the questions and trained the enumerators to avoid or reduce personal bias during data collection. The three hundred twenty-three SHF selected using systematic random sampling (based on their livestock and pastureland potential) from eight Kebeles (23 from Genatit, 39 from Fitto, 44 from Deferge, 20 from Dedere, 29 from Cheleme, 48 from Yamed, 77 from Kurkur, and 43 from Kibtiya) were individually interviewed to collect both the qualitative and quantitative data (Fig 1). We collected data from April through June 2024. The quatitative data included livestock numbers and the size of land uses, and the other items. Triangulations of information obtained from questionnaire interview were done by FGDs in each Kebele.

### Data analysis

Excel spreadsheet 2022 was employed to organize the collected original data. We checked, coded, and validated the normality or completeness of the collected data before statistical analysis. Then, the data were subjected to SAS software version 9.0 [54], and descriptive statistics such as frequencies, percentages, standard deviations, and means were provided for interpretation whenever found appropriate. A figure and tables were used to present the results. In addition, a non-parametric inferential analysis, specifically rank regression at *p* < 0.05, was conducted to examine the direction and strength of the relationship between socio-economic attributes and pastureland use types as sources of IHF, the reasons for the decline of natural pasture, and techniques for managing natural pasture (Table 1). The rank regression analysis yielded outputs such as coefficients, standard errors, t-values, and p-values for socio-economic attributes associated with each dependent variable.

**Table 1:** Description of dependent and independent variables considered for rank regression analysis.

Variables		Descriptions
Dependent	Grazing land use types	Private grazing, open grazing, roadside, and fallow land were ranked 1–4
	Reasons for the decline of natural pasture	Cropland expansion, urbanization, bush encroachment, overgrazing, soil erosion, and forestland expansion were ranked 1–6
	Techniques of natural pasture management	Flood water, irrigation, killing rodents, manure application, zero grazing, deferred grazing, and reduce animals were considered during the dry season and ranked 1–7. Weed removal, urea fertilizer application, manure application, zero grazing, and reduce animals were considered techniques used during the wet season and ranked 1–5.
Independent	Sex	1 = Female, 2 = Male
	Age	1 = < 30 years old, 2 = 30–45 years old, 3 = > 45 years old
	Educational status	1 = Illiterate, 2 = Read and write, 3 = Elementary school, 4 = High school, 5 = Preparatory school, 6 = College, 7 = University
	Marita status	1 = Single, 2 = Married, 3 = Divorced, 4 = widow

Moreover, when the descriptive analysis dictated the existence of a statistically significant difference between Districts for continuous variables, mean comparisons were carried out using the t-test at *p* < 0.05, applying the following statistical model:


$$\begin{array}{c} \text{Yijk}=\mu +\text{Si}+\text{eijk} \end{array}$$ (4)

Where Y_ijk_ = household variables; µ = overall mean; S_i_ = the effect of i^th^ District (i = Tenta and Mekdela Districts); e_ijk_ = the random error.

Data such as major sources of feed, seasonal utilization of animal feeds, land use types as sources of IHGLS, reasons for the decline of natural pasture, and challenges and mitigation options in utilizing IHGLS were summarized using the priority index formula. The following index (*I*) formula was applied with the principle of weighted average developed by Musa et al. [55]:


$$\begin{array}{c} \text{I}=\frac{Rn*C\text{1}+Rn-\text{1}*C\text{2}+\ldots +R\text{1}*Cn}{\sum Rn*C\text{1}+Rn-\text{1}*C\text{2}+\ldots +R\text{1}*Cn} \end{array}$$ (5)

Where, Rn = Value given for the least ranked level. Cn = Counts of the least ranked level

## Results and discussion

### Socio-economic characteristics of the respondents

Table 2 summarizes socio-economic characteristics of the respondents at District levels. The study included 323 farmers with extensive knowledge of feeds, natural pasture, and IHGLS, of whom 89.16% were male and 10.84% were female. More than half of the respondents (53.5%) were in the 30–45 years age category, which agreed with the results of Yesuph and Dagnew [47]. Moreover, the respondents had diverse educational statuses, with the majority being illiterate (29.1%) and able to read and write (28.79%). Ninety percent of the respondents were married.

**Table 2:** Socio-economic attributes of respondents in the study area.

Attributes		District (n = 323)		Overall n (%)
		Makidela	Tenta	
Sex	Female	29 (22.31%)	6 (3.11%)	35 (10.84)
	Male	101 (77.69%)	187 (96.89%)	288 (89.16%)
Age category	<30 years	21 (16.15%)	5 (2.59%)	26 (8.05%)
	30-45 years	70 (53.85%)	102 (52.85%)	172 (53.25%)
	>45 years	39 (30%)	86 (44.56%)	125 (38.7%)
Educational background	Illiterate	43 (33.08%)	51 (26.42%)	94 (29.1%)
	Read and write	34 (26.15%)	59 (30.57%)	93 (28.79%)
	Elementary school (1–8)	37 (28.46%)	44 (22.8%)	81 (25.08%)
	Secondary school (9–10)	10 (7.69%)	19 (9.84%)	29 (8.98%)
	Preparatory school (11–12)	4 (3.08%)	19 (9.84%)	23 (7.13%)
	College	1 (0.77)		1 (0.31%)
	University	1 (0.77%)	1 (0.52%)	2 (0.62%)
Marital status	Divorced	11 (8.46%)	7 (3.63%)	18 (5.57%)
	Married	110 (84.62%)	183 (94.82%)	293 (90.71%)
	Single	5 (3.85%)	1 (0.52%)	6 (1.86%)
	Widow	4 (3.08%)	2 (1.04%)	6 (1.86%)
Total		130 (40.25%)	193 (59.75%)	323 (100%)

Table 3 presents the household family size and animal population size of the respondents. The t-test analysis yielded no significant difference (*p* > 0.05) for many variables between the Mekdela and Tenta Districts, except for local sheep, crossbred cattle, exotic cattle, and donkeys. Tenta District (4.58 ± 1.36) had a relatively higher family size per household than Mekdela (4.44 ± 1.53), which is in line with the results of a previous study by Yesuph and Dagnew [47]. Tenta District had relatively higher crossbred sheep (4.17 ± 3.6), local chicken (2.99 ± 1.49), exotic chicken (3.04 ± 1.54), local cattle (2.74 ± 1.17), and modern bee colonies (2 ± 2) at the household level compared to Mekdela District.

**Table 3:** Family size and animal population size of the respondents.

Variables		Districts (n = 323)				Overall		p-value
		Mekdela (n = 130)		Tenta (n = 193)				
		Mean	SD	Mean	SD	Mean	SD	
Family size	Male	2.36	1.14	2.28	1.08	2.31	1.1	0.5414
	Female	2.18	0.98	2.32	1.14	2.26	1.08	0.2791
	Total	4.44	1.53	4.58	1.36	4.52	1.43	0.3828
Animal Population	Local sheep	7.72^b^	4.74	10.21^a^	6.38	9.25	5.92	0.0006
	Cross-bred sheep	3.3	2.37	4.17	3.6	3.83	3.19	0.2711
	Exotic sheep	1	–	1	–	1	0	0.8822
	Local chicken	2.89	1.65	2.99	1.49	2.95	1.56	0.7231
	Exotic chicken	3	1.52	3.04	1.57	3.02	1.55	0.8822
	Cross-bred chicken	2.46	1.37	2	1	2.36	1.28	0.6051
	Local cattle	2.47	1.36	2.74	1.17	2.64	1.25	0.1032
	Cross-bred cattle	2.12^b^	1.14	2.92^a^	1.92	2.54	1.65	0.0010
	Exotic cattle	1.46^a^	0.69	1^b^	0	1.22	0.52	0.0321
	Goats	1.83	1.01	1.53	0.77	1.63	0.87	0.0535
	Donkey	1.56^a^	0.66	1.4^b^	0.57	1.46	0.61	0.0194
	Horse	1.07	0.26	1.02	0.14	1.04	0.2	0.2807
	Mule	1.14	0.38	1	–	1.03	0.18	0.0629
	Traditional bee colony	2.5	3.33	1.84	1.09	2.06	2.09	0.2752
	Transitional bee colony	2	1	–	–	1.5	0.71	–
	Modern bee colony	1.8	1.3	2	2	1.89	1.54	0.8608

### Trend in total livestock population and reasons for declining

Table 4 shows the animal population statuses and different reasons for its decline, in which nearly half of smallholder farmers (50.26%) in Tenta District reduced their animals compared to farmers in Mekdela District (40%). Considerable proportion of respondents (46.13%) in both Districts indicated that most farmers decreased their animal population size, mainly pertaining to a decline in natural pasture and feed scarcity. This is in line with the study of Yesuph and Dagnew [47], who noted that the livestock number per household had been declining with time, pertaining to land shortage for forage production. The large majority of respondents perceived that the occurrence of drought, decline in natural pasture, and feed scarcity were the main reasons for the reduction of animal population from year to year. Similarly, different works approved the reduction of livestock population in different areas of Ethiopia attributable to a decline in natural pasture, climate change, and overgrazing [7,14]. In addition, Begna et al. [56] and Policy Studies Institute of Ethiopia [57] confirmed that the decline in pastureland, frequent drought occurrence and conventional farming techniques are the main causes of decline in livestock population across Northeastern Ethiopia. The same trends in livestock population were observed in Northeastern Africa (Sudan, Eritrea, and Somalia) and Northeastern Asia (Mongolia and China), but the main reasons, including climate change, overgrazing, desertification, frequent conflict, and displacement in Northeastern Africa, and urbanisation and land use change in Northeastern Asia, were significantly different [58].

**Table 4:** Trends of livestock holding and possible reasons of declining status based on perceptions of respondents.

Variables		Districts				
		Mekdela (n = 130)		Tenta (n = 193)		Total respondents n (%)
		N	%	N	%	
Trend in animal population	Decreasing	52	40	97	50.26	149 (46.13)
	Increasing	44	33.85	59	30.57	103 (31.89)
	Stable	29	22.31	29	15.03	58 (17.9)
	Unknown	5	3.85	8	4.15	13 (4.02)
Reasons for declining	Decline in natural pasture	9	17.31	17	17.71	26 (17.57)
	Decline in natural pasture and drought	1	1.92	4	4.17	5 (3.38)
	Drought	4	7.69	7	7.29	11 (7.43)
	Feed scarcity	9	17.31	18	18.75	27 (18.25)
	Feed scarcity and decline in natural pasture	4	7.69	18	18.75	22 (14.86)
	Feed scarcity and drought	1	1.92	7	7.29	8 (5.41)
	Feed scarcity, decline in natural pasture, and drought	7	13.46	12	12.5	19 (12.84)
	Decline in natural pasture, invaded weed and drought			1	1.04	1 (0.68)
	lack of labor	1	1.92	2	2.08	3 (2.03)
	All	16	30.77	10	10.42	26 (17.57)

### Major land use types

Table 5 shows that communal grazing land (4.85 ± 2.23 ha) occupied the largest land area for animal production, followed by cropland (0.5 ± 0.18 ha) and private grazing land (0.484 ± 0.16 ha). A study conducted in the upper Beshillo catchments of Tenta District revealed that most farmers have 0.5 ha of cropland and 0.25 ha of pastureland [47]. It is also supported by Girma et al. [59] and Talore [42], who stated that natural pasture and crop residue constitute the fundamental portion of animal feeds in different parts of Ethiopia. Communal pastureland is the predominant feed source in northeastern Ethiopia, though a lack of appropriate management leads to serious degradation [60]. Overgrazing of overgrazed communal lands was frequently observed in the study area, which is in agreement with the studies of Talore [42]. Though there was variation among smallholder farmers for all land use types in the study area, significant differences were not found (*p* > 0.05), except for private pasture land (*p* < 0.0029). Forest, fallow, improved forage, and roadside lands had a minute contribution to animal feeds in the study area. This might be attributable to the government having been expanding area exclosures for a long time to conserve soil and water. A similar trend was observed in Northeastern Ethiopia and Northeastern Africa (Somalia, Kenya, and Sudan), in which the available pastureland has declined due to the expansion of exclosure areas [61] and overutilization [62,63]. Moreover, the carrying capacity of pasturelands in the globe, particularly Asia and Europe, has been reduced to 27%, mainly due to overgrazing [64].

**Table 5:** Land use types in ha used for livestock feed as per the respondents knowledge (n = 323).

Land use type	Districts							
	Mekdela (n = 130)		Tenta (n = 193)		Overall (n = 323)		p-value	CV (%)
	Mean	SD	Mean	SD	Mean	SD		
Cropland	0.5	0.2	0.5	0.17	0.502	0.18	0.8617	35.93
Private pastureland	0.48	0.14	0.48	0.17	0.484	0.158	0.0029	32.6
Forestland	0.2	0.06	0.2	0.07	0.2	0.065	0.5370	32.75
Farrow land	0.16	0.06	0.2	0.06	0.189	0.063	0.2601	33.42
Improved forage land	0.07	0.04	0.06	0.05	0.064	0.046	0.5243	71.88
Communal grazing land	4.84	2.26	4.85	2.21	4.848	2.227	0.9539	45.95
Road side grazing land	0.1	0	0.1	0.02	0.101	0.012	0.2456	11.68

### Major sources of livestock feed, and importance of natural pasture

Table 6 summarizes sources of animal feed, the main use of natural pasture, predominant utilization practices of natural pasture, and farmers’ plans to utilize the natural pasture. Farmers used natural pasture mainly as animal feed (98.74%). Similarly, the wider northeastern Ethiopian highlands provide 80% of the livestock feed from natural pasture [65], whereas in developed regions of the northeastern world, cultivated forage and zero grazing of natural pasture were dominant [66]. More than half of the respondents (52.8%) gained animal feed from on-farm and communal grazing, followed by purchased, farm products, and communal grazing (38.51%), which is in line with the work done in northern Ethiopia [67]. The study found that the large majority of farmers (88.85%) had experience in natural pasture utilization. Global trends showed that bold determination is required to integrate pastureland and livestock population for sustainable utilization [66], and tools such as Global Pasture Watch help to follow up on the condition and health of pasturelands [68]. Many respondents (81.42%) predominantly applied both medium grazing and the cut-and-carry system, followed by both free grazing and the cut-and-carry system (7.12%), which is consistent with the reports of FAO [69]. In line with the present study, Taddese et al. [70] suggested that a cut-and-carry system could optimize the productivity of natural pasture. Moreover, the implementation of medium grazing had been considered the best option to sustain the livestock sector [15,71]. In contrast to the results of the present study, Gurmessa [72] noted that among all smallholder farmers in Eastern Ethiopia, only 17.3% of farmers have practiced the cut-and-carry system.

**Table 6:** Perception of respondents on sources of animal feeds, experience in natural pasture utilization and plans (n = 323).

Variables		Districts n (%)		
		Mekdela	Tenta	Overall
Source of animal feed	On-farm	6 (4.65)	9 (4.66)	15 (4.66)
	On-farm and purchased	2 (1.55)	4 (2.07)	6 (1.86)
	On-farm and communal grazing	66 (51.16)	104 (53.89)	170 (52.8)
	Purchased and communal grazing	3 (2.33)	4 (2.07)	7 (2.17)
	Purchased, on-farm and communal grazing	52 (40.31)	72 (37.3)	124 (38.51)
Main use of NP	As animal feed	125 (99.21)	187 (98.42)	312 (98.74)
	As source of income	1 (0.79)	3 (1.58)	4 (1.27)
Experience in NPU	Yes	118 (90.77)	169 (87.56)	287 (88.85)
	No	12 (9.23)	24 (12.44)	36 (11.15)
Predominant utilization practices	Free grazing	3 (2.31)	7 (3.63)	10 (3.1)
	Medium grazing and cut and carry		2 (1.04)	2 (0.62)
	both medium grazing and cut and carry	95 (73.08)	168 (87.05)	263 (81.42)
	free grazing and zero grazing	13 (10)	10 (5.18)	23 (7.12)
	medium grazing	5 (3.85)	1 (0.52)	6 (1.86)
	no grazing/cut and carry	14 (10.77)	5 (2.59)	19 (5.88)
Intention to expand GL	Yes	19 (14.62)	16 (8.29)	35 (10.84)
	No	111 (85.38)	177 (91.71)	288 (89.16)
Future plan	Cut and carry	1 (1.82)	3 (3.57)	4 (2.88)
	both medium grazing and cut and carry	21 (38.18)	24 (28.57)	45 (32.27)
	medium grazing	1 (1.82)	–	1 (0.72)
	no grazing/cut and carry	32 (58.18)	57 (67.86)	89 (64.03)

GL: Grazing Land; NPU: Natural Pasture Utilization.

Many farmers (89.2%) had no intention to expand their grazing land in the study area. More interestingly, all farmers planned to stop free grazing, and the majority (64.03%) would practice the cut-and-carry system, followed by both medium grazing and the cut-and-carry system (32.27%). Different stakeholders, such as productive safety net programs, are encouraged to apply zero grazing to all pasturelands because heavy grazing could result in land degradation [70–75].

Natural pasture remains the major and cheap source of livestock feed in Ethiopia [15,76], which agrees with the results of the present study. In this regard, the present study suggests community-based grazing land management as the best option, particularly for areas with a medium population size, to alleviate feed shortages and sustain the use of natural pasture. In line with the aforementioned recommendation, around 90% of villages in Ethiopia had at least one protected pastureland covering, on average, 38.2 ha, where farmers could have multipurpose uses [77]. Because protection of natural pasture from livestock resulted in greater above- and below-ground carbon concentrations and hence improved forage production [31,78]. However, heavily grazed pastureland requires a longer recovery period to provide the maximum herbage biomass [24], which agrees with the perception of farmers in the study area.

### Seasonal utilization of animal feeds

Table 7 shows the ranking of available feed resources by respondents during the dry and wet seasons. Farmers in the study area ranked hay (*I* = 0.279) as the main source of animal feed throughout the year, followed by crop residue (*I* = 0.24) and natural pasture (*I* = 0.182), which aligns with previous studies [59,79]. The increase in crop residue and other feed sources reflects the reduction of pastureland by 9.3% from 1973 to 2011 [22]. During the dry season, the available feeds ranked third to eighth were natural pasture, concentrates (wheat bran, soybean meal, noug seed cake, brewer’s spent grain, and molasses), green forage, non-conventional feed, improved forage, and weed, respectively. Farmers mainly drive crop residue and weeds from wheat, barley, beans, peas, and lentils. Additionally, improved forages like tree lucern and Acacia saligna, along with green forage and hay from natural pasture and backyards, were used as the primary sources of animal feed in the study area. Similarly, the scarcity of feed forced northeastern Africa to use non-conventional feeds [80]. In addition, the globe focused on insects and food waste to implement precise animal nutrition for sustainable profit [81,82].

**Table 7:** Ranking of feed availability during the dry and wet seasons as per the respondents perception (n = 323).

Variables		Ranks								Index	Overall rank	Year round	
		1^st^	2^nd^	3^rd^	4^th^	5^th^	6^th^	7^th^	8^th^			Index	rank
Dry season	Crop residue	52	234	37						0.253	2	0.24	2
	Improved forage		15	32	10	4				0.04	7	0.032	7
	Natural pasture		17	221	67	11	5			0.205	3	0.182	3
	Concentrates		2	11	69	56	20	1		0.079	4	0.054	6
	Hay	271	47							0.278	1	0.279	1
	Green forage			6	68	46	8	11		0.068	5	0.064	5
	Non-conventional feed			6	34	32	18			0.043	6	0.031	8
	Weed			2	25	23	17	5	11	0.034	8	0.118	4
	Total	323	315	315	273	172	68	17	11				
Wet season	Crop residue	25	137	96	60	5				0.227	2		
	Improved forage			7	20	18	4	1		0.023	7		
	Natural pasture	2	40	62	104	53	32	20		0.16	4		
	Concentrates			2	24	32	7			0.028	6		
	Hay	257	43	13	6					0.281	1		
	Green forage	3	10	35	35	20				0.06	5		
	Non-conventional feed			4	7	18	21	3		0.019	8		
	Weed	36	93	104	46	10				0.202	3		
	Total	323	323	323	302	156	64	24					

### Sources of indigenous herbaceous forages

The present study highlights the overall ranking of land use types and their relation to socio-economic attributes as sources of IHF for farmers in the study area (Table 8). Age (*p* = 0.0078) and marital status (*p* = 0.0087) had a significant and positive relation to roadside and fallow land as sources of IHF. In agreement with this result, Abaynew et al. [83] reported that the SHF who were married and older were likely to practice conventional grazing systems in roadside and fallow land. Moreover, household structure and age affect access to land and sources of feed in northeastern Africa [63]. However, the impact of age and marital status is not significant globally where mechanised and market-oriented feeds are available [84].

**Table 8:** Relative importance of grazing land use types as sources of indigenous herbaceous forages and their association with socio-economic attributes.

Land use types	Socio-economic attributes	Coefficient	St. Error	t-Value	P-Value	Ranks (n = 323)				Index	Overall rank
						1^st^	2^nd^	3^rd^	4^th^		
Private pasture	Sex	0.07	0.05	1.39	0.1648	294	22			0.433	1
	Age	−0.004	0.03	−0.13	0.8985						
	Education	0.001	0.03	0.03	0.9722						
	Marriage	−0.062	0.05	−1.24	0.2158						
Open grazing land	Sex	−0.06	0.05	−1.09	0.2772	25	275	3		0.325	2
	Age	−0.01	0.03	−0.24	0.8111						
	Education	−0.02	0.03	−0.62	0.5340						
	Marriage	0.07	0.06	1.1	0.2734						
Road side	Sex	0.01	0.06	0.16	0.8769	3	19	282	19	0.228	3
	Age	0.104	0.04	2.68	0.0078						
	Education	0.038	0.04	1.09	0.2775						
	Marriage	−0.036	0.07	−0.55	0.5840						
Fallow land	Sex	0.01	0.01	1.01	0.3296		1	19		0.014	4
	Age	0.003	0.01	0.37	0.7198						
	Education	−0.003	0.01	−0.45	0.6569						
	Marriage	0.03	0.01	3.01	0.0087						
Total						322	317	304	19		

Respondents reported that private pastureland (*I* = 0.433) was the primary source of IHF in the study area, followed by open grazing (*I* = 0.325), whereas fallow land (I = 0.014) contributed less. Similarly, in the wider northeastern Ethiopia, private pastureland is becoming dominant following the policy shift to reduce communal pastureland while increasing exclosure areas [61]. In contrast, communal pastureland is still the dominant land use practice in northeastern Africa, though private pastureland is expanding following reforms done on land tenure [62]. In addition, private pastureland dominates the livestock feed in northeastern China, but roadsides and fallow land were not considered, as they brought pollution to the environment [85]. Moreover, according to Phillips [86] and Faber-Langendoen and Josse [87], natural pasture in highlands is the main source of plant diversity, which agrees with the results of the present study. Generally, the abundance of grass and legume species in the natural pasture has reduced over time due to continuous grazing, persistent drought, population pressure, and expansion of cropland, bushland, and weeds [13,71,88,89–91].

### Farmers experience of natural pasture improvement

The majority of respondents (53.87%) had no experience in natural pasture management in the study area (Table 9), which is in agreement with the results of previous work by Talore [42]. Respondents (70.9%) believed that the natural pasture had been decreasing with time, whereas 22.9% and 6.2% of sampled farmers stated that the natural pasture was stable and its status was unknown, respectively. The conversion of natural pasture into cropland and bushland [18,22], soil erosion [23], and heavy grazing [71,88] due to population pressure caused a significant feed deficit. The survey carried out in Ethiopia indicated that the poor rangeland condition is primarily due to overgrazing, followed by the occurrence of drought, human population pressure, immigration, a lack of knowledge, and poor soil conditions [92].

**Table 9:** Experiences in natural pasture management and challenges in indigenous herbaceous forage development.

Attributes		Districts (n = 323)					
		Mekdela (n = 130)		Tenta (n = 193)		Overall	
		N	%	N	%	N	%
Challenges in IHF	No	20	15.38	29	15.03	49	15.17
	Yes	110	84.62	164	84.97	274	84.83
Experience PLM	No	73	56.15	101	52.33	174	53.87
	Yes	57	43.84	92	47.67	149	46.13
Natural pasture status	Decreasing	78	60	151	78.24	229	70.9
	Stable	49	37.69	25	12.95	74	22.9
	Unknown	3	2.31	17	8.81	20	6.2

PLM: Pasture Land Management; IHF: Indigenous Herbaceous Forage; n: number of respondents.

Furthermore, researchers noted the positive contribution of utilizing the culturally rich indigenous knowledge and practice in Africa to adapt to the existing climate [93] and emphasize its bold role in sustaining development issues [94]. In addition, other works highlighted that indigenous knowledge magnifies the importance of science, and their integration plays a critical role in natural resource management while enhancing adaptation to the changing climate, especially where modern technology is absent [95,96]. Smallholder farmers (85%) in Ethiopia have a deep understanding of their environment and the existing change, including the climate, to make informed decisions and ensure their agricultural practices are sustainable and resilient [95]. However, Aticho et al. [97] reported that farmers in the Jima Zone, Ethiopia, had less factual knowledge of their environment. Furthermore, Mugambiwa [98] and Mekonnen et al. [95] suggested the presence of traditional organizations to make the community aware and implement sustainable and effective land use programs at reduced failure of adaptation to climate change. Therefore, indigenous knowledge is a wealth accumulated over a long time in response to challenges, including climate change, and should be considered adequately in policy and strategy development to use natural pasture efficiently and sustainably.

Table 10 presents the ranking of reasons for the decline of natural pasture and their association with socio-economic attributes in the study area. Age (coefficient = −0.15; *p* < 0.0001) had a significant adverse association with urbanization, in which older SHF perceived urbanization as the main cause of decline in natural pasture compared to the youth. Sex (coefficient = −0.2; *p* = 0.0374) and marital status (coefficient = −0.21; *p* = 0.0413) had a strong and negative relation to forestland expansion, which means SHF who were male and widowed expanded forestland, which reduced natural pasture, highlighting the importance of gender to manage and access grazing land resources in northeastern Ethiopia [83]. Forestland expansion (*I* = 0.456) was the first and most outstanding reason, followed by cropland expansion (*I* = 0.338), which is consistent with the results obtained from the wider northeastern Ethiopia [61], northeastern Africa [63], and northeastern China [85]. This could be attributable to the extent to which they implement soil and conservation strategies following the government’s attention to increasing forest cover in different parts of the country [60]. After the implementation of soil and water conservation structures, the local people strengthened their work with biological options and applied community by-laws to prevent grazing. In addition, Proclamation No. 456/2005, art. 13.3 of the Ethiopian federal government, particularly the Federal Rural Land Administration and Land Use, prohibited continuous grazing, aiming to conserve soil and water and ultimately leading to a decline of animal population [9,99]. Sinore et al. [100] promoted biological measures of soil and water conservation methods to support the livestock with adequate forage, which agrees with what was observed in the study area.

**Table 10:** Reasons for the decline of natural pasture and their relation to socio-economic attributes.

Reasons (dependent variables)	Predictor	Coefficient	St. Error	t-Value	P-Value	Ranks						Index	Overall rank
						1^st^	2^nd^	3^rd^	4^th^	5^th^	6^th^		
Crop land expansion	Sex	0.1	0.07	1.52	0.1299	147	47	15		3		0.338	2
	Age	0.02	0.04	0.54	0.5884								
	Education	−0.08	0.04	−1.93	0.0548								
	Marriage	0.13	0.07	1.77	0.0779								
Urbanization	Sex	0.03	0.06	0.49	0.6242	13	26	57	16		1	0.139	3
	Age	−0.15	0.04	−4.39	<0.0001								
	Education	−0.02	0.03	−0.46	0.6487								
	Marriage	0.07	0.06	1.05	0.2963								
Bush encroachment	Sex	−0.003	0.01	−0.44	0.6888			6		1		0.007	5
	Age	0.006	0.01	1.07	0.3621								
	Education	0.013	0.01	2.48	0.0890								
	Marriage	0	−	−	−								
Over grazing	Sex	−0.01	0.03	−0.34	0.7387	16	18	3	2			0.058	4
	Age category	−0.01	0.02	−0.47	0.6411								
	Education	0.03	0.02	1.53	0.1362								
	Marriage status	0.01	0.04	0.13	0.9000								
Soil erosion	Sex	0	–	–	–			1				0.001	6
	Age	0	–	–	–								
	Education	0	–	–	–								
	Marriage	0	–	–	–								
Forest land expansion	Sex	−0.2	0.096	−2.09	0.0374	134	146	15				0.456	1
	Age	−0.04	0.057	−0.68	0.4952								
	Education	0.05	0.051	0.95	0.3413								
	Marriage	−0.21	0.101	−2.05	0.0413								

Mengistu et al. [101] and Gurmessa [72] stated that resettlement and cultivation have reduced the size of natural pasture, which agrees with the result in the present study. Urbanization, overgrazing, bush encroachment, and erosion were ranked third, fourth, fifth, and sixth by their contribution to shrinking natural pasture in the study area. According to the study of Yesuph and Dagnew [47], one-third of the natural pasture at the Gedallas watershed had been declining between 1986 and 2017. The same author demonstrated that those farmers listed overgrazing, the expansion of cropland, and resettlement as the major causes of land use change. In 1973, grassland covered 19, 19.6, and 30% of the Meiso, Tiyo, and Liben districts, respectively, but in 2007, it covered 11, 4.33, and 7.33% of those respective districts [22] in Ethiopia. This result highlighted the rapid loss of grassland by 42.11–75.57% over 34 years, which is in line with the present study. Enkossa et al. [26] also noted the current rapid conversion of grazing land into different landscapes in Ethiopia over the last 45 years, since 1973.

### Challenges in indigenous herbaceous forages

The majority of the respondents (84.83%) noticed the presence of challenges in utilizing the IHF in the study area (Table 11). The other 15.17% of sampled farmers had no experience in identifying the challenges of utilizing IHF. Farmers listed and ranked 10 challenges of utilizing IHGLS in the study area. Land shortage (*I* = 0.172), lack of awareness (*I* = 0.17), and eucalyptus expansion (*I* = 0.151) were identified as the three most influential challenges of utilizing IHGLS. Similar reports were noted for the devastating influence of inadequate land and the expansion of eucalyptus trees [61] and the limited awareness level [83] of the loss of natural pasture in northeastern Ethiopia. Farmers also cited climate change, free grazing, poor production potential, and lack of appropriate technology as the challenges of utilizing IHGLS in the study area. Erratic and unreliable rainfall, together with the increase in temperature, degraded the pastureland potential while increasing its sensitivity to climate change [49]. Moreover, their unavailability, lack of adequate capital, and inadequate market access limited the potential utilization of IHF, which is in agreement with the works of Tesfay et al. [102]. In contrast, earlier studies identified expansion of bush encroachment and cropland as the most common constraints to utilizing the IHGLS in natural pasture in Ethiopia [22,74] pertaining to different agro-ecologies and production systems. Moreover, the same authors also identified constraints such as overgrazing, drought, direction change of the river, lack of knowledge, little government attention, expansion of towns, increase in human population, immigration, poor soil status, and conversion into forestland. Bush encroachment greatly deteriorates the natural pasture, and its control is part of the prior program to optimize the productivity of rangelands in Ethiopia [75].

**Table 11:** Challenges to utilizing indigenous herbaceous grass and legume forage species.

Attributes	Ranks										Index	Overall rank
	1^st^	2^nd^	3^rd^	4^th^	5^th^	6^th^	7^th^	8^th^	9^th^	10^th^		
Lack of awareness	91	153	24								0.17	2
Poor availability					2		2	158	94		0.047	8
Free grazing		3	16	84	163						0.118	5
Lack of appropriate technology					2	78	188				0.079	7
Land shortage	171	53	30	8	3	3					0.172	1
Climate change				176	68	19	1	2	2		0.12	4
Lack of capital							2	104	124		0.039	9
Inadequate market access								6	40	149	0.017	10
Poor production potential			3		20	169	74	2			0.088	6
Eucalyptus expansion	9	56	195		8						0.151	3
Total	271	265	268	268	266	269	267	272	260	149		

### Methods of managing natural pasture during the dry and wet seasons

Table 12 and Table 13 present the index and rankings of methods of improving the IHF species in the natural pastureland and their association with socio-economic attributes. Only killing rodents was strongly adversely related to marital status (coefficient = −0.2; *p* = 0.0051) during the dry season, in which widowed respondents killed few rodents to improve their natural pasture (Table 12). During the wet season, age (coefficient = −0.04; *p* = 0.0243) and education status (coefficient = −0.08; *p* = 0.0355) had a negative and significant influence on the application of manure and zero grazing, respectively, indicating older and educated SHF were less likely to implement the respective techniques to properly improve the natural pasture. On the other hand, education status (coefficient = 0.08; *p* = 0.0250) had a significant positive impact on reducing the number of animals during the wet season (Table 13), suggesting educated farmers were more likely to decide to reduce the herd size. Reports in the broader northeastern Ethiopia indicated that older and illiterate farmers depend largely on conventional grazing land utilization [83], which is consistent with the present study. A work done in northeastern Africa indicated that education supports applying sustainable herd and pastureland management [62].

**Table 12:** Techniques of natural pasture management and their relationship to socio-economic attributes to utilize indigenous herbaceous forages during the dry season.

Dependent variable	Socio-economic attributes	Coefficient	St. Error	t-Value	P-Value	Ranks							Index	Overall Rank
						1^st^	2^nd^	3^rd^	4^th^	5^th^	6^th^	7^th^		
Flood water	Sex	−0.02	0.08	−0.29	0.772				1	77	58		0.09	5
	Age category	0.02	0.04	0.55	0.5843									
	Education	−0.01	0.04	−0.4	0.6901									
	Marriage status	−0.01	0.07	−0.16	0.8714									
Irrigation	Sex	−0.01	0.08	−0.15	0.8821	53	54			2			0.179	4
	Age category	−0.001	0.04	−0.03	0.9757									
	Education	0.02	0.03	0.63	0.5268									
	Marriage status	−0.04	0.06	−0.72	0.4753									
Killing rodents	Sex	0.1	0.07	1.38	0.1703		2			2	46	40	0.038	7
	Age category	0.01	0.03	0.37	0.7120									
	Education	0.03	0.03	0.97	0.3344									
	Marriage status	−0.2	0.07	−2.87	0.0051									
Manure application	Sex	0.04	0.06	0.77	0.4423			7	31	57			0.084	6
	Age category	0.03	0.03	0.97	0.3341									
	Education	−0.02	0.03	−0.54	0.5908									
	Marriage status	0.04	0.06	0.71	0.4791									
Zero grazing	Sex	0.07	0.08	0.87	0.3850	16	23	32	78				0.184	3
	Age category	−0.03	0.04	−0.81	0.4192									
	Education	−0.04	0.04	−1.01	0.3163									
	Marriage status	0.11	0.07	1.48	0.1403									
Deferred grazing	Sex	0.05	0.08	0.59	0.5576	22	9	74	42				0.191	2
	Age category	0.004	0.04	0.08	0.9333									
	Education	−0.02	0.04	−0.63	0.5306									
	Marriage status	−0.08	0.08	−1.06	0.2908									
Reduce animals	Sex	−0.01	0.08	−0.09	0.9272	55	61	33					0.234	1
	Age category	0.01	0.04	0.24	0.8106									
	Education	−0.01	0.04	−0.3	0.7621									
	Marriage status	0.07	0.08	0.98	0.3285									

**Table 13:** Techniques of natural pasture management and their relationship to socio-economic factors to utilize indigenous herbaceous forages during the wet season.

Dependent variable	Socio-economic attributes	Coefficient	St. Error	t-Value	P-Value	Ranks					Index	Overall Rank
						1^st^	2^nd^	3^rd^	4^th^	5^th^		
Weed removal	Sex	−0.002	0.06	−0.04	0.9707		3	10	44	28	0.08	5
	Age category	0.02	0.03	0.68	0.4990							
	Educational status	−0.03	0.03	−0.97	0.3370							
	Marriage status	0.005	0.07	0.07	0.9422							
Urea fertilizer application	Sex	0.02	0.06	0.26	0.7975			26	57	3	0.1	4
	Age category	−0.02	0.03	−0.64	0.5249							
	Educational status	−0.04	0.03	−1.38	0.1704							
	Marriage status	−0.01	0.05	−0.25	0.8016							
Manure application	Sex	−0.01	0.04	−0.25	0.8047	1	7	98			0.17	3
	Age category	−0.04	0.02	−2.29	0.0243							
	Educational status	−0.01	0.02	−0.29	0.7747							
	Marriage status	0.03	0.03	0.97	0.3364							
Zero grazing	Sex	−0.08	0.08	−1.11	0.2694	80	69				0.34	1
	Age category	−0.03	0.04	−0.78	0.4365							
	Educational status	−0.08	0.04	−2.12	0.0355							
	Marriage status	−0.01	0.07	−0.12	0.9040							
Reduce animal number	Sex	0.08	0.07	1.07	0.2875	68	73				0.32	2
	Age category	0.02	0.04	0.52	0.6065							
	Educational status	0.08	0.04	2.27	0.0250							
	Marriage status	0.02	0.07	0.26	0.7961							

The ranking of techniques of natural pasture management by local farmers shows that herd size (*I* = 0.234) is the first in its contribution during the dry season, followed by employing deferred grazing (*I* = 0.191) and zero grazing (*I* = 0.184). This is in line with earlier studies of Abule et al. [74] and Mekasha et al. [22], who identified reducing the number of livestock as the primary mitigation option. On the other hand, farmers ranked stop-free grazing (*I* = 0.34) as the primary method of natural pasture management during the wet season in the study area, followed by reducing animals (*I* = 0.32). Similarly, pastoralists perceived alternatives such as irrigation practices, deferred grazing, and reducing animal numbers as the keys to improving their rangeland productivity [92]. Farmers controlled rodents in cultivated land in northeastern Africa, but killing them in pastureland is not commonly applied [63]. The application of manure and zero grazing are recommended in Kenya and Uganda [80]. Alemayehu et al. [15] suggested that pasture rest in August through November increased herbage production. Grazing exclusion improves soil nitrogen [32] and soil carbon [103] and ultimately optimizes pastureland productivity [18,23,33,47]. In addition, it is noted that 95% of smallholder farmers gained high-quality feed by preventing the communal pastureland from free grazing [72]. On the other hand, the study conducted in the central highlands of Ethiopia suggested medium grazing for optimum biomass production of forage from IHF [46]. Similar to the results of the present study, Worqlul et al. [104] suggested small-scale irrigation to provide adequate and quality feed for their animals.

## Conclusion

In conclusion, the smallholder farmers have deep-rooted indigenous knowledge about major land uses that provide forage for animals, feed utilization, and challenges and methods of improving local herbaceous forages in the natural pasture. In the study area, livestock production is mainly dependent on natural pasture. However, the natural pasture has been declining with time at an alarming rate, mainly due to forestland, cropland, and resettlement expansion. Land shortage, lack of awareness, and eucalyptus expansion are also becoming challenges in utilizing indigenous herbaceous forages in the northeastern Ethiopian highlands. Farmers have practiced different methods of improving the indigenous herbaceous forages in the natural pasture, particularly reducing animal numbers and zero grazing during the dry and wet seasons, respectively. Furthermore, age, education, and marital status determine the methods to be applied to manage and utilize natural pasturelands. Therefore, the policy-makers should show bold determination to include the indigenous knowledge to use and manage the natural pasture effectively and sustainably. However, the study was limited to two Districts due to time and financial constraints, though the northeastern Ethiopian highlands are diverse in agro-ecology and socio-economic attributes. Moreover, additional studies should be carried out to identify the indigenous herbaceous forage species that are abundant, palatable, productive, resistant to grazing, and tolerant to drought to provide high-quality forage for animals.

## Supporting information

S1 File Raw data. (XLS)
