# Supplementary material for: Farmers’ indigenous knowledge on local herbaceous forages in the Northeastern highlands of Ethiopia
Source: PLoS One. 2026 Jun 26;21(6):e0352188. doi: 10.1371/journal.pone.0352188 (PMC13308867; doi:10.1371/journal.pone.0352188)
Supplement: Attachment — Submitted filename: Response to Reviewers.docx [file pone.0352188.s003.docx]

Response to Decision Letter to the Journal of PLOS ONE

Manuscript ID: PONE-D-25-40748

Manuscript Title: Farmers’ indigenous knowledge on local herbaceous forages in the Northeastern highlands of Ethiopia

Dear Academic Editor and Reviewers,

Thank you for giving us the opportunity to submit a revised version of our manuscript entitled “Farmers’ indigenous knowledge on local herbaceous forages in the Northeastern highlands of Ethiopia” to the PLOS ONE Journal. We are very grateful to the academic editor and reviewers for their highly relevant comments, which helped us, improve our manuscript comprehensively to meet the scientific standard and address a wider audience. We prepared our response below to address all issues commented on by the academic editor and reviewers, as requested, and highlighted the changes within the manuscript with track changes. We are happy to receive further comments, thank you in advance.

Academic Editor’s comments

**Comment: 1)** The author should strengthen the theoretical framework to highlight how the findings advance knowledge on indigenous practices and climate adaptation,

**Response:** We highly thank you, the editor, for your relevant comment. We revised the manuscript as per your suggestion.

**Comment: 2)** supplement descriptive statistics with inferential analyses (e.g., regression) to explore socio-economic variable relationships,

**Response:** We are so grateful for this very important comment. We did a non-parametric inferential analysis, specifically rank regression at p < 0.05, and determined the direction and strength of the relationship between socio-economic attributes and pastureland use types as sources of IHF, the reasons for the decline of natural pasture, and techniques for managing natural pasture. The rank regression analysis yielded outputs such as coefficients, standard errors, t-values, and p-values for socio-economic attributes associated with each dependent variable. The details of the parameter were presented in Table 1.

**Comment: 3)** provide details on ethical approval and informed consent,

**Response:** Thank you very much for your relevant comment to provide ethical approval and informed consent details. We addressed these issues in the Material and methods of the revised manuscript on page 9, lines 148 to 153.

**Comment: 4)** deposit data in a public repository to meet full open-access requirements, and avoid result repetition; focus on interpretation, global literature comparisons, and policy implications.

**Response:** We appreciate the academic editor’s comment. We have fixed these issues in the revised version of the manuscript. In doing so, we addressed more than 20 articles and used them in our manuscript; as a result, we added 20 additional reference lists. We will upload the data as Supporting Information files. Regarding policy implications, we highlighted the relevance of considering indigenous knowledge while preparing national policy related to natural pasture resource management and improvement.

Review Comments to the Author

Reviewer #1:

**Comment: 1)** This manuscript reported the farmers’ knowledge about local herbaceous forages from two districts of northeastern highlands of Ethiopia. The study is important and authors employ a well-structured analytical approach using survey data, however, following are some aspects, which should be addressed for improved presentation and scientific integrity of the manuscript.

**Response:** We thank you, dear reviewer, for your deep and relevant comments.

**Comment: 2)** L 22. Define SHF

**Response:** We appreciate the reviewer's comment. Therefore, we corrected the concern in the revised version of the manuscript, as smallholder farmers (SHF).

Introduction

**Comment: 3)** Objectives are clear and scientific background is provided.

**Response:** Thank you for your grateful comment.

**Comment: 4)** Keeping in view the wide diversity in agroecology, culture, socioeconomics, and farmer knowledge, it is uncertain that the selected two districts truly represent all the northeastern highlands as mentioned in the title. Therefore, it is necessary to be properly justified in introduction about generalization to the wider northeastern highlands.

**Response:** We thank you and appreciate the reviewer's concern to strengthen the clarity of this study. We selected the two Districts based on their natural pasture area coverage, livestock population and socio-economic attributes, as it was clearly written in Materials and methods of the revised version of the manuscript on pages 6 and 7, line numbers 104 to 111. Recognizing the heterogeneity of the northeastern highlands of Ethiopia, the selected districts provide meaningful representation of the area so that they can provide relevant information across similar areas.

Methodology

**Comment: 5)** L 78. What is the average rainfall and temperature of overall northeastern highlands.

**Response:** We thank you, the reviewer, for your help to strengthen the clarity and vigor of this manuscript. The highlands of northeastern Ethiopia predominantly have an annual temperature and an annual rainfall ranging from 12°C to 28°C and 800 mm to 1200 mm, respectively, with an increase in annual temperature of 0.07°C [82-84].

**Comment: 6)** Please mention the approval of study by the relevant authority?

**Response:** Thank you, dear reviewer, for your valuable comment. We mentioned the relevant authority that approved the study on page 9, line numbers 152 and 153.

**Comment: 7)** L 96. Check for capitalization of the first letter within sentences.

**Response:** Thank you, we have corrected it in the revised manuscript.

**Comment: 8)** Was the questionnaire structured and validated?

**Response:** Thank you for your insightful comment. We used a structured and semi-structured questionnaire, which was validated.

**Comment: 9)** It is mentioned that multistage sampling procedure was adapted, however, relevant details on how respondents were selected at each stage are not included.

**Response:** Thank you for your helpful comment to enhance the reproducibility of the study. We have addressed your concern in Material and method of the revised version of the manuscript on page 7 line number 115 to 117.

**Comment: 10)** As mentioned in abstract primary and secondary data, how secondary data compliment the primary data.

**Response:** Thank you for your relevant comment. We have corrected it in the revised version of the manuscript.

Results and discussion

**Comment: 11)** Please discuss this data in comparison with wider northeastern highlands.

**Response:** We acknowledge the reviewer. We compare the present findings with the broader northeastern highlands of Ethiopian, African, and the globe.

**Comment: 12)** Mention the details about types of feed. For example, what are different types of crop residues, improved forages, hay, green forages, and weed etc. presented in table 6.

**Response:** Thank you, dear reviewer, for your grateful suggestion. We have included your concern in the revised version of the manuscript on page 20 line number 296 to 301.

**Comment: 13)** Add some practical strategies at the end to cope with the shortage problem in addition to future directions.

**Response:** Thank you for your helpful comments. we considered your comment in Tables 12 and 13

**Comment: 14)** Limitations of the study needs to be acknowledged in conclusion.

**Response:** Thank you for your meaningful comment. We addressed it in the conclusion part of the revised manuscript.

Reviewer #2

**Comment: 1)** This paper explores farmers’ indigenous knowledge of local herbaceous forages in Ethiopia, a relevant and valuable topic. The study provides rich empirical data. However, several issues need addressing.

**Response:** Thank you, dear reviewer, for your grateful comments.

**Comment: 2)** The manuscript mainly presents descriptive findings. The discussion does not sufficiently highlight how the study advances existing theories on indigenous knowledge, sustainable pasture management, or climate adaptation. The authors should strengthen the theoretical framing and explain how their results contribute to broader debates.

**Response:** We are so grateful for these very important and constructive comments. We did a non-parametric inferential analysis, specifically rank regression at p < 0.05, and determined the direction and strength of the relationship between socio-economic attributes and pastureland use types as sources of IHF, the reasons for the decline of natural pasture, and techniques for managing natural pasture. The rank regression analysis yielded outputs such as coefficients, standard errors, t-values, and p-values for socio-economic attributes associated with each dependent variable. The details of the parameter were presented in Table 1. In addition, we explicitly connected the present finding s with the broader northeastern highlands of Ethiopia, Africa, and the world.

**Comment: 3)** Although the sample size (n=323) is appropriate, the representativeness of the two districts should be discussed. Moreover, the analysis is heavily descriptive. Applying inferential statistics (e.g., regression models, factor analysis) could provide deeper insights into relationships between socio-economic variables and forage management practices.

**Response:** We appreciated the comments and included them in the revised version of the manuscript as discussed in the previous comments.

**Comment: 4)** The manuscript currently reports “N/A,” but because the study involved household interviews and focus groups, details about ethical approval and informed consent are necessary. At minimum, state whether institutional or local approval was obtained and clarify how consent was secured.

**Response:** We acknowledged the reviewer comments and included in the revised version of the manuscript under the Materials and methods on page 9 line number 148 to 153.

**Comment: 5)** PLOS ONE requires full open access to data. The current statement (“available upon request”) does not meet the policy. Authors should deposit data in a public repository or clearly justify restrictions.

**Response:** We appreciate the reviewer's comment. We will upload the data as Supporting Information files.

**Comment: 6)** The discussion section repeats many results. More emphasis should be placed on interpretation, comparison with international literature, and implications for policy and practice. Connecting the Ethiopian case with global studies on traditional ecological knowledge would increase impact.

**Response:** We appreciate the reviewer comments. We have fixed these issues in the revised version of the manuscript. In doing so, we addressed more than 20 articles and used them in our manuscript; as a result, we added 20 additional reference lists.
