# Supplementary material for: Farmers’ indigenous knowledge on local herbaceous forages in the Northeastern highlands of Ethiopia
Source: PLoS One. 2026 Jun 26;21(6):e0352188. doi: 10.1371/journal.pone.0352188 (PMC13308867; doi:10.1371/journal.pone.0352188)
Supplement: Peer review history

# Peer review: Decision Letter 0

**DOI:** 10.1371/journal.pone.0352188.r001

**Authors:** Abebayehu Aticho

1 Sep 2025

PONE-D-25-40748

Farmers’ indigenous knowledge on local herbaceous forages in the Northeastern highlands of Ethiopia

PLOS ONE

Dear Dr. Ebrahim,

Thank you for submitting your manuscript to PLOS ONE. After careful consideration, we feel that it has merit but does not fully meet PLOS ONE’s publication criteria as it currently stands. Therefore, we invite you to submit a revised version of the manuscript that addresses the points raised during the review process.

The author should: (1) strengthen the theoretical framework to highlight how the findings advance knowledge on indigenous practices and climate adaptation, (2) supplement descriptive statistics with inferential analyses (e.g., regression) to explore socio-economic variable relationships, (3) provide details on ethical approval and informed consent, (4)  deposit data in a public repository to meet full open-access requirements, and avoid result repetition; focus on interpretation, global literature comparisons, and policy implications. Failling to doing these would lead to rejection of the manuscript.

Please submit your revised manuscript by Oct 16 2025 11:59PM. If you will need more time than this to complete your revisions, please reply to this message or contact the journal office at plosone@plos.org. When you're ready to submit your revision, log on to https://www.editorialmanager.com/pone/ and select the 'Submissions Needing Revision' folder to locate your manuscript file.

Please include the following items when submitting your revised manuscript:

- A rebuttal letter that responds to each point raised by the academic editor and reviewer(s). You should upload this letter as a separate file labeled 'Response to Reviewers'.
- A marked-up copy of your manuscript that highlights changes made to the original version. You should upload this as a separate file labeled 'Revised Manuscript with Track Changes'.
- An unmarked version of your revised paper without tracked changes. You should upload this as a separate file labeled 'Manuscript'.

If you would like to make changes to your financial disclosure, please include your updated statement in your cover letter. Guidelines for resubmitting your figure files are available below the reviewer comments at the end of this letter.

If applicable, we recommend that you deposit your laboratory protocols in protocols.io to enhance the reproducibility of your results. Protocols.io assigns your protocol its own identifier (DOI) so that it can be cited independently in the future. For instructions see: https://journals.plos.org/plosone/s/submission-guidelines#loc-laboratory-protocols. Additionally, PLOS ONE offers an option for publishing peer-reviewed Lab Protocol articles, which describe protocols hosted on protocols.io. Read more information on sharing protocols at https://plos.org/protocols?utm_medium=editorial-email&utm_source=authorletters&utm_campaign=protocols.

We look forward to receiving your revised manuscript.

Kind regards,

Abebayehu Aticho

Academic Editor

PLOS ONE

Journal requirements:

When submitting your revision, we need you to address these additional requirements.

1. Please ensure that your manuscript meets PLOS ONE's style requirements, including those for file naming. The PLOS ONE style templates can be found at

https://journals.plos.org/plosone/s/file?id=wjVg/PLOSOne_formatting_sample_main_body.pdf and

https://journals.plos.org/plosone/s/file?id=ba62/PLOSOne_formatting_sample_title_authors_affiliations.pdf

2. Please provide additional details regarding participant consent. In the ethics statement in the Methods and online submission information, please ensure that you have specified (1) whether consent was informed and (2) what type you obtained (for instance, written or verbal, and if verbal, how it was documented and witnessed). If your study included minors, state whether you obtained consent from parents or guardians. If the need for consent was waived by the ethics committee, please include this information.

If you are reporting a retrospective study of medical records or archived samples, please ensure that you have discussed whether all data were fully anonymized before you accessed them and/or whether the IRB or ethics committee waived the requirement for informed consent. If patients provided informed written consent to have data from their medical records used in research, please include this information.

3. We note that you have indicated that there are restrictions to data sharing for this study. For studies involving human research participant data or other sensitive data, we encourage authors to share de-identified or anonymized data. However, when data cannot be publicly shared for ethical reasons, we allow authors to make their data sets available upon request. For information on unacceptable data access restrictions, please see http://journals.plos.org/plosone/s/data-availability#loc-unacceptable-data-access-restrictions.

Before we proceed with your manuscript, please address the following prompts:

a) If there are ethical or legal restrictions on sharing a de-identified data set, please explain them in detail (e.g., data contain potentially identifying or sensitive patient information, data are owned by a third-party organization, etc.) and who has imposed them (e.g., a Research Ethics Committee or Institutional Review Board, etc.). Please also provide contact information for a data access committee, ethics committee, or other institutional body to which data requests may be sent.

b) If there are no restrictions, please upload the minimal anonymized data set necessary to replicate your study findings to a stable, public repository and provide us with the relevant URLs, DOIs, or accession numbers. Please see http://www.bmj.com/content/340/bmj.c181.long for guidelines on how to de-identify and prepare clinical data for publication. For a list of recommended repositories, please see https://journals.plos.org/plosone/s/recommended-repositories. You also have the option of uploading the data as Supporting Information files, but we would recommend depositing data directly to a data repository if possible.

Please update your Data Availability statement in the submission form accordingly.

4. Please amend either the title on the online submission form (via Edit Submission) or the title in the manuscript so that they are identical.

5. Please amend the manuscript submission data (via Edit Submission) to include author Yeshambel Mekuriaw, Bimrew Asmare, Netsanet Beyero, Fentahun Meheret Zeleke, Shewaye Hailecherkos, Endalew Mekonen, Zerihun Nigussie, Wubetie Adnew.

6. Please include a separate caption for each figure in your manuscript.

7. We note that Figure 1 in your submission contain [map/satellite] images which may be copyrighted. All PLOS content is published under the Creative Commons Attribution License (CC BY 4.0), which means that the manuscript, images, and Supporting Information files will be freely available online, and any third party is permitted to access, download, copy, distribute, and use these materials in any way, even commercially, with proper attribution. For these reasons, we cannot publish previously copyrighted maps or satellite images created using proprietary data, such as Google software (Google Maps, Street View, and Earth). For more information, see our copyright guidelines: http://journals.plos.org/plosone/s/licenses-and-copyright.

We require you to either (1) present written permission from the copyright holder to publish these figures specifically under the CC BY 4.0 license, or (2) remove the figures from your submission:

1. You may seek permission from the original copyright holder of Figure(s) [#] to publish the content specifically under the CC BY 4.0 license.

We recommend that you contact the original copyright holder with the Content Permission Form (http://journals.plos.org/plosone/s/file?id=7c09/content-permission-form.pdf) and the following text:

“I request permission for the open-access journal PLOS ONE to publish XXX under the Creative Commons Attribution License (CCAL) CC BY 4.0 (http://creativecommons.org/licenses/by/4.0/). Please be aware that this license allows unrestricted use and distribution, even commercially, by third parties. Please reply and provide explicit written permission to publish XXX under a CC BY license and complete the attached form.”

Please upload the completed Content Permission Form or other proof of granted permissions as an "Other" file with your submission.

In the figure caption of the copyrighted figure, please include the following text: “Reprinted from [ref] under a CC BY license, with permission from [name of publisher], original copyright [original copyright year].”

2. If you are unable to obtain permission from the original copyright holder to publish these figures under the CC BY 4.0 license or if the copyright holder’s requirements are incompatible with the CC BY 4.0 license, please either i) remove the figure or ii) supply a replacement figure that complies with the CC BY 4.0 license. Please check copyright information on all replacement figures and update the figure caption with source information. If applicable, please specify in the figure caption text when a figure is similar but not identical to the original image and is therefore for illustrative purposes only.

The following resources for replacing copyrighted map figures may be helpful:

USGS National Map Viewer (public domain): http://viewer.nationalmap.gov/viewer/

The Gateway to Astronaut Photography of Earth (public domain): http://eol.jsc.nasa.gov/sseop/clickmap/

Maps at the CIA (public domain): https://www.cia.gov/library/publications/the-world-factbook/index.html and https://www.cia.gov/library/publications/cia-maps-publications/index.html

NASA Earth Observatory (public domain): http://earthobservatory.nasa.gov/

Landsat: http://landsat.visibleearth.nasa.gov/

USGS EROS (Earth Resources Observatory and Science (EROS) Center) (public domain): http://eros.usgs.gov/#

Natural Earth (public domain): http://www.naturalearthdata.com/

8. If the reviewer comments include a recommendation to cite specific previously published works, please review and evaluate these publications to determine whether they are relevant and should be cited. There is no requirement to cite these works unless the editor has indicated otherwise.

Additional Editor Comments (if provided):

Reviewer #1:

Reviewer #2:

[Note: HTML markup is below. Please do not edit.]

Reviewers' comments:

Reviewer's Responses to Questions

**Comments to the Author**

1. Is the manuscript technically sound, and do the data support the conclusions?

The manuscript must describe a technically sound piece of scientific research with data that supports the conclusions. Experiments must have been conducted rigorously, with appropriate controls, replication, and sample sizes. The conclusions must be drawn appropriately based on the data presented.

Reviewer #1: Partly

Reviewer #2: Partly

**********

2. Has the statistical analysis been performed appropriately and rigorously?

Reviewer #1: Yes

Reviewer #2: N/A

**********

3. Have the authors made all data underlying the findings in their manuscript fully available?

The PLOS Data policy requires authors to make all data underlying the findings described in their manuscript fully available without restriction, with rare exception (please refer to the Data Availability Statement in the manuscript PDF file). The data should be provided as part of the manuscript or its supporting information, or deposited to a public repository. For example, in addition to summary statistics, the data points behind means, medians and variance measures should be available. If there are restrictions on publicly sharing data—e.g. participant privacy or use of data from a third party—those must be specified.

Reviewer #1: Yes

Reviewer #2: No

**********

4. Is the manuscript presented in an intelligible fashion and written in standard English?

PLOS ONE does not copyedit accepted manuscripts, so the language in submitted articles must be clear, correct, and unambiguous. Any typographical or grammatical errors should be corrected at revision, so please note any specific errors here.

Reviewer #1: Yes

Reviewer #2: Yes

**********

5. Review Comments to the Author

Please use the space provided to explain your answers to the questions above. You may also include additional comments for the author, including concerns about dual publication, research ethics, or publication ethics. (Please upload your review as an attachment if it exceeds 20,000 characters)

Reviewer #1: This manuscript reported the farmers knowledge about local herbaceous forages from two districts of northeastern highlands of Ethiopia. The study is important and authors employ a well-structured analytical approach using survey data, however, following are some aspects, which should be addressed for improved presentation and scientific integrity of the manuscript.

L 22. Define SHF

Introduction

Objectives are clear and scientific background is provided.

Keeping in view the wide diversity in agroecology, culture, socioeconomics, and farmer knowledge, it is uncertain that the selected two districts truly represent all the northeastern highlands as mentioned in the title. Therefore, it is necessary to be properly justified in introduction about generalization to the wider northeastern highlands.

Methodology

L 78. What is the average rainfall and temperature of overall northeastern highlands.

Please mention the approval of study by the relevant authority?

L 96. Check for capitalization of the first letter within sentences.

Was the questionnaire structured and validated?

It is mentioned that multistage sampling procedure was adapted, however, relevant details on how respondents were selected at each stage are not included.

As mentioned in abstract primary and secondary data, how secondary data compliment the primary data.

Results and discussion

Please discuss this data in comparison with wider northeastern highlands.

Mention the details about types of feed. For example, what are different types of crop residues, improved forages, hay, green forages, and weed etc. presented in table 6.

Add some practical strategies at the end to cope with the shortage problem in addition to future directions.

Limitations of the study needs to be acknowledged in conclusion.

Reviewer #2: This paper explores farmers’ indigenous knowledge of local herbaceous forages in Ethiopia, a relevant and valuable topic. The study provides rich empirical data. However, several issues need addressing.

1.The manuscript mainly presents descriptive findings. The discussion does not sufficiently highlight how the study advances existing theories on indigenous knowledge, sustainable pasture management, or climate adaptation. The authors should strengthen the theoretical framing and explain how their results contribute to broader debates.

2.Although the sample size (n=323) is appropriate, the representativeness of the two districts should be discussed. Moreover, the analysis is heavily descriptive. Applying inferential statistics (e.g., regression models, factor analysis) could provide deeper insights into relationships between socio-economic variables and forage management practices.

3.The manuscript currently reports “N/A,” but because the study involved household interviews and focus groups, details about ethical approval and informed consent are necessary. At minimum, state whether institutional or local approval was obtained and clarify how consent was secured.

4.PLOS ONE requires full open access to data. The current statement (“available upon request”) does not meet the policy. Authors should deposit data in a public repository or clearly justify restrictions.

5.The discussion section repeats many results. More emphasis should be placed on interpretation, comparison with international literature, and implications for policy and practice. Connecting the Ethiopian case with global studies on traditional ecological knowledge would increase impact.

**********

6. PLOS authors have the option to publish the peer review history of their article (what does this mean?). If published, this will include your full peer review and any attached files.

If you choose “no”, your identity will remain anonymous but your review may still be made public.

**Do you want your identity to be public for this peer review?**  For information about this choice, including consent withdrawal, please see our Privacy Policy.

Reviewer #1: No

Reviewer #2: No

**********

[NOTE: If reviewer comments were submitted as an attachment file, they will be attached to this email and accessible via the submission site. Please log into your account, locate the manuscript record, and check for the action link "View Attachments". If this link does not appear, there are no attachment files.]

While revising your submission, please upload your figure files to the Preflight Analysis and Conversion Engine (PACE) digital diagnostic tool, https://pacev2.apexcovantage.com/. PACE helps ensure that figures meet PLOS requirements. To use PACE, you must first register as a user. Registration is free. Then, login and navigate to the UPLOAD tab, where you will find detailed instructions on how to use the tool. If you encounter any issues or have any questions when using PACE, please email PLOS at figures@plos.org. Please note that Supporting Information files do not need this step.

# Author comment: Author response to Decision Letter 1

**DOI:** 10.1371/journal.pone.0352188.r002

17 Nov 2025

Response to Decision Letter to the Journal of PLOS ONE

Manuscript ID: PONE-D-25-40748

Manuscript Title: Farmers’ indigenous knowledge on local herbaceous forages in the Northeastern highlands of Ethiopia

Dear Academic Editor and Reviewers,

Thank you for giving us the opportunity to submit a revised version of our manuscript entitled “Farmers’ indigenous knowledge on local herbaceous forages in the Northeastern highlands of Ethiopia” to the PLOS ONE Journal. We are very grateful to the academic editor and reviewers for their highly relevant comments, which helped us, improve our manuscript comprehensively to meet the scientific standard and address a wider audience. We prepared our response below to address all issues commented on by the academic editor and reviewers, as requested, and highlighted the changes within the manuscript with track changes. We are happy to receive further comments, thank you in advance.

Academic Editor’s comments

Comment: 1) The author should strengthen the theoretical framework to highlight how the findings advance knowledge on indigenous practices and climate adaptation,

Response: We highly thank you, the editor, for your relevant comment. We revised the manuscript as per your suggestion.

Comment: 2) supplement descriptive statistics with inferential analyses (e.g., regression) to explore socio-economic variable relationships,

Response: We are so grateful for this very important comment. We did a non-parametric inferential analysis, specifically rank regression at p < 0.05, and determined the direction and strength of the relationship between socio-economic attributes and pastureland use types as sources of IHF, the reasons for the decline of natural pasture, and techniques for managing natural pasture. The rank regression analysis yielded outputs such as coefficients, standard errors, t-values, and p-values for socio-economic attributes associated with each dependent variable. The details of the parameter were presented in Table 1.

Comment: 3) provide details on ethical approval and informed consent,

Response: Thank you very much for your relevant comment to provide ethical approval and informed consent details. We addressed these issues in the Material and methods of the revised manuscript on page 9, lines 148 to 153.

Comment: 4) deposit data in a public repository to meet full open-access requirements, and avoid result repetition; focus on interpretation, global literature comparisons, and policy implications.

Response: We appreciate the academic editor’s comment. We have fixed these issues in the revised version of the manuscript. In doing so, we addressed more than 20 articles and used them in our manuscript; as a result, we added 20 additional reference lists. We will upload the data as Supporting Information files. Regarding policy implications, we highlighted the relevance of considering indigenous knowledge while preparing national policy related to natural pasture resource management and improvement.

Review Comments to the Author

Reviewer #1:

Comment: 1) This manuscript reported the farmers’ knowledge about local herbaceous forages from two districts of northeastern highlands of Ethiopia. The study is important and authors employ a well-structured analytical approach using survey data, however, following are some aspects, which should be addressed for improved presentation and scientific integrity of the manuscript.

Response: We thank you, dear reviewer, for your deep and relevant comments.

Comment: 2) L 22. Define SHF

Response: We appreciate the reviewer's comment. Therefore, we corrected the concern in the revised version of the manuscript, as smallholder farmers (SHF).

Introduction

Comment: 3) Objectives are clear and scientific background is provided.

Response: Thank you for your grateful comment.

Comment: 4) Keeping in view the wide diversity in agroecology, culture, socioeconomics, and farmer knowledge, it is uncertain that the selected two districts truly represent all the northeastern highlands as mentioned in the title. Therefore, it is necessary to be properly justified in introduction about generalization to the wider northeastern highlands.

Response: We thank you and appreciate the reviewer's concern to strengthen the clarity of this study. We selected the two Districts based on their natural pasture area coverage, livestock population and socio-economic attributes, as it was clearly written in Materials and methods of the revised version of the manuscript on pages 6 and 7, line numbers 104 to 111. Recognizing the heterogeneity of the northeastern highlands of Ethiopia, the selected districts provide meaningful representation of the area so that they can provide relevant information across similar areas.

Methodology

Comment: 5) L 78. What is the average rainfall and temperature of overall northeastern highlands.

Response: We thank you, the reviewer, for your help to strengthen the clarity and vigor of this manuscript. The highlands of northeastern Ethiopia predominantly have an annual temperature and an annual rainfall ranging from 12°C to 28°C and 800 mm to 1200 mm, respectively, with an increase in annual temperature of 0.07°C [82-84].

Comment: 6) Please mention the approval of study by the relevant authority?

Response: Thank you, dear reviewer, for your valuable comment. We mentioned the relevant authority that approved the study on page 9, line numbers 152 and 153.

Comment: 7) L 96. Check for capitalization of the first letter within sentences.

Response: Thank you, we have corrected it in the revised manuscript.

Comment: 8) Was the questionnaire structured and validated?

Response: Thank you for your insightful comment. We used a structured and semi-structured questionnaire, which was validated.

Comment: 9) It is mentioned that multistage sampling procedure was adapted, however, relevant details on how respondents were selected at each stage are not included.

Response: Thank you for your helpful comment to enhance the reproducibility of the study. We have addressed your concern in Material and method of the revised version of the manuscript on page 7 line number 115 to 117.

Comment: 10) As mentioned in abstract primary and secondary data, how secondary data compliment the primary data.

Response: Thank you for your relevant comment. We have corrected it in the revised version of the manuscript.

Results and discussion

Comment: 11) Please discuss this data in comparison with wider northeastern highlands.

Response: We acknowledge the reviewer. We compare the present findings with the broader northeastern highlands of Ethiopian, African, and the globe.

Comment: 12) Mention the details about types of feed. For example, what are different types of crop residues, improved forages, hay, green forages, and weed etc. presented in table 6.

Response: Thank you, dear reviewer, for your grateful suggestion. We have included your concern in the revised version of the manuscript on page 20 line number 296 to 301.

Comment: 13) Add some practical strategies at the end to cope with the shortage problem in addition to future directions.

Response: Thank you for your helpful comments. we considered your comment in Tables 12 and 13

Comment: 14) Limitations of the study needs to be acknowledged in conclusion.

Response: Thank you for your meaningful comment. We addressed it in the conclusion part of the revised manuscript.

Reviewer #2

Comment: 1) This paper explores farmers’ indigenous knowledge of local herbaceous forages in Ethiopia, a relevant and valuable topic. The study provides rich empirical data. However, several issues need addressing.

Response: Thank you, dear reviewer, for your grateful comments.

Comment: 2) The manuscript mainly presents descriptive findings. The discussion does not sufficiently highlight how the study advances existing theories on indigenous knowledge, sustainable pasture management, or climate adaptation. The authors should strengthen the theoretical framing and explain how their results contribute to broader debates.

Response: We are so grateful for these very important and constructive comments. We did a non-parametric inferential analysis, specifically rank regression at p < 0.05, and determined the direction and strength of the relationship between socio-economic attributes and pastureland use types as sources of IHF, the reasons for the decline of natural pasture, and techniques for managing natural pasture. The rank regression analysis yielded outputs such as coefficients, standard errors, t-values, and p-values for socio-economic attributes associated with each dependent variable. The details of the parameter were presented in Table 1. In addition, we explicitly connected the present finding s with the broader northeastern highlands of Ethiopia, Africa, and the world.

Comment: 3) Although the sample size (n=323) is appropriate, the representativeness of the two districts should be discussed. Moreover, the analysis is heavily descriptive. Applying inferential statistics (e.g., regression models, factor analysis) could provide deeper insights into relationships between socio-economic variables and forage management practices.

Response: We appreciated the comments and included them in the revised version of the manuscript as discussed in the previous comments.

Comment: 4) The manuscript currently reports “N/A,” but because the study involved household interviews and focus groups, details about ethical approval and informed consent are necessary. At minimum, state whether institutional or local approval was obtained and clarify how consent was secured.

Response: We acknowledged the reviewer comments and included in the revised version of the manuscript under the Materials and methods on page 9 line number 148 to 153.

Comment: 5) PLOS ONE requires full open access to data. The current statement (“available upon request”) does not meet the policy. Authors should deposit data in a public repository or clearly justify restrictions.

Response: We appreciate the reviewer's comment. We will upload the data as Supporting Information files.

Comment: 6) The discussion section repeats many results. More emphasis should be placed on interpretation, comparison with international literature, and implications for policy and practice. Connecting the Ethiopian case with global studies on traditional ecological knowledge would increase impact.

Response: We appreciate the reviewer comments. We have fixed these issues in the revised version of the manuscript. In doing so, we addressed more than 20 articles and used them in our manuscript; as a result, we added 20 additional reference lists.

Attachment Submitted filename: Response to Reviewers.docx

# Peer review: Decision Letter 1

**DOI:** 10.1371/journal.pone.0352188.r003

**Authors:** Abebayehu Aticho, Jianhong Zhou

7 Jun 2026

<p>Farmers’ indigenous knowledge on local herbaceous forages in the Northeastern highlands of Ethiopia

PONE-D-25-40748R1

Dear Dr. Ebrahim,

We’re pleased to inform you that your manuscript has been judged scientifically suitable for publication and will be formally accepted for publication once it meets all outstanding technical requirements.

Within one week, you’ll receive an e-mail detailing the required amendments. When these have been addressed, you’ll receive a formal acceptance letter and your manuscript will be scheduled for publication.

An invoice will be generated when your article is formally accepted. Please note, if your institution has a publishing partnership with PLOS and your article meets the relevant criteria, all or part of your publication costs will be covered. Please make sure your user information is up-to-date by logging into Editorial Manager at Editorial Manager® and clicking the ‘Update My Information' link at the top of the page. For questions related to billing, please contact billing support.

If your institution or institutions have a press office, please notify them about your upcoming paper to help maximize its impact. If they’ll be preparing press materials, please inform our press team as soon as possible -- no later than 48 hours after receiving the formal acceptance. Your manuscript will remain under strict press embargo until 2 pm Eastern Time on the date of publication. For more information, please contact onepress@plos.org.

Kind regards,

Jianhong Zhou

Staff Editor

PLOS One

Additional Editor Comments (optional):

Reviewers' comments:

Reviewer's Responses to Questions

**Comments to the Author**

1. If the authors have adequately addressed your comments raised in a previous round of review and you feel that this manuscript is now acceptable for publication, you may indicate that here to bypass the “Comments to the Author” section, enter your conflict of interest statement in the “Confidential to Editor” section, and submit your "Accept" recommendation.

Reviewer #1: All comments have been addressed

Reviewer #2: (No Response)

**********

2. Is the manuscript technically sound, and do the data support the conclusions?

The manuscript must describe a technically sound piece of scientific research with data that supports the conclusions. Experiments must have been conducted rigorously, with appropriate controls, replication, and sample sizes. The conclusions must be drawn appropriately based on the data presented.

Reviewer #1: Yes

Reviewer #2: Yes

**********

3. Has the statistical analysis been performed appropriately and rigorously?

Reviewer #1: Yes

Reviewer #2: Yes

**********

4. Have the authors made all data underlying the findings in their manuscript fully available?

The PLOS Data policy requires authors to make all data underlying the findings described in their manuscript fully available without restriction, with rare exception (please refer to the Data Availability Statement in the manuscript PDF file). The data should be provided as part of the manuscript or its supporting information, or deposited to a public repository. For example, in addition to summary statistics, the data points behind means, medians and variance measures should be available. If there are restrictions on publicly sharing data—e.g. participant privacy or use of data from a third party—those must be specified.

Reviewer #1: Yes

Reviewer #2: Yes

**********

5. Is the manuscript presented in an intelligible fashion and written in standard English?

PLOS ONE does not copyedit accepted manuscripts, so the language in submitted articles must be clear, correct, and unambiguous. Any typographical or grammatical errors should be corrected at revision, so please note any specific errors here.

Reviewer #1: Yes

Reviewer #2: Yes

**********

6. Review Comments to the Author

Please use the space provided to explain your answers to the questions above. You may also include additional comments for the author, including concerns about dual publication, research ethics, or publication ethics. (Please upload your review as an attachment if it exceeds 20,000 characters)

Reviewer #1: (No Response)

Reviewer #2: The authors have addressed all previous reviewer and editor comments. The revised version demonstrates substantial improvement in scientific rigor, analytical depth, and clarity. Both the theoretical framework and data interpretation now meet the standards for publication in PLOS ONE. The manuscript is coherent, well-organized, and makes a meaningful contribution to the understanding of indigenous knowledge systems and sustainable pasture management in Ethiopia.

This revised manuscript now fulfills PLOS ONE’s scientific, ethical, and open-access standards. The topic is regionally significant and internationally relevant, contributing valuable empirical insights into the intersection of indigenous knowledge and pasture ecology.

**********

7. PLOS authors have the option to publish the peer review history of their article (what does this mean?). If published, this will include your full peer review and any attached files.

If you choose “no”, your identity will remain anonymous but your review may still be made public.

**Do you want your identity to be public for this peer review?**  For information about this choice, including consent withdrawal, please see our Privacy Policy.

Reviewer #1: No

Reviewer #2: No

**********

# Editor report: Acceptance letter

**DOI:** 10.1371/journal.pone.0352188.r004

**Authors:** Abebayehu Aticho, Jianhong Zhou

PONE-D-25-40748R1

PLOS One

Dear Dr. Ebrahim,

I'm pleased to inform you that your manuscript has been deemed suitable for publication in PLOS One. Congratulations! Your manuscript is now being handed over to our production team.

At this stage, our production department will prepare your paper for publication. This includes ensuring the following:

* All references, tables, and figures are properly cited

* All relevant supporting information is included in the manuscript submission,

* There are no issues that prevent the paper from being properly typeset

You will receive further instructions from the production team, including instructions on how to review your proof when it is ready. Please keep in mind that we are working through a large volume of accepted articles, so please give us a few days to review your paper and let you know the next and final steps.

Lastly, if your institution or institutions have a press office, please let them know about your upcoming paper now to help maximize its impact. If they'll be preparing press materials, please inform our press team within the next 48 hours. Your manuscript will remain under strict press embargo until 2 pm Eastern Time on the date of publication. For more information, please contact onepress@plos.org.

You will receive an invoice from PLOS for your publication fee after your manuscript has reached the completed accept phase. If you receive an email requesting payment before acceptance or for any other service, this may be a phishing scheme. Learn how to identify phishing emails and protect your accounts at https://explore.plos.org/phishing.

If we can help with anything else, please email us at customercare@plos.org.

Thank you for submitting your work to PLOS ONE and supporting open access.

Kind regards,

PLOS ONE Editorial Office Staff

on behalf of

Dr. Jianhong Zhou

Staff Editor

PLOS One
